# Perturbation-based balance training: Principles, mechanisms and implementation in clinical practice

**DOI:** 10.3389/fspor.2022.1015394

**Published:** 2022-10-06

**Authors:** Christopher McCrum, Tanvi S. Bhatt, Marissa H. G. Gerards, Kiros Karamanidis, Mark W. Rogers, Stephen R. Lord, Yoshiro Okubo

**Affiliations:** ^1^Department of Nutrition and Movement Sciences, NUTRIM School of Nutrition and Translational Research in Metabolism, Maastricht University, Maastricht, Netherlands; ^2^Neuromotor Rehabilitation Research Group, Department of Rehabilitation Sciences, KU Leuven, Leuven, Belgium; ^3^Department of Physical Therapy, College of Applied Health Sciences, University of Illinois, Chicago, IL, United States; ^4^Department of Epidemiology, Care and Public Health Institute (CAPHRI), Maastricht University, Maastricht, Netherlands; ^5^Department of Physiotherapy, Maastricht University Medical Center (MUMC+), Maastricht, Netherlands; ^6^Sport and Exercise Science Research Centre, School of Applied Sciences, London South Bank University, London, United Kingdom; ^7^Department of Physical Therapy and Rehabilitation Science, School of Medicine, University of Maryland, Baltimore, MD, United States; ^8^Falls, Balance and Injury Research Centre, Neuroscience Research Australia, Sydney, NSW, Australia; ^9^Faculty of Medicine and Health, University of New South Wales, Sydney, NSW, Australia

**Keywords:** aged, slips, trips, gait adaptation, balance disorders, rehabilitation, accidental falls

## Abstract

Since the mid-2000s, perturbation-based balance training has been gaining interest as an efficient and effective way to prevent falls in older adults. It has been suggested that this task-specific training approach may present a paradigm shift in fall prevention. In this review, we discuss key concepts and common issues and questions regarding perturbation-based balance training. In doing so, we aim to provide a comprehensive synthesis of the current evidence on the mechanisms, feasibility and efficacy of perturbation-based balance training for researchers and practitioners. We address this in two sections: “Principles and Mechanisms” and “Implementation in Practice.” In the first section, definitions, task-specificity, adaptation and retention mechanisms and the dose-response relationship are discussed. In the second section, issues related to safety, anxiety, evidence in clinical populations (e.g., Parkinson's disease, stroke), technology and training devices are discussed. Perturbation-based balance training is a promising approach to fall prevention. However, several fundamental and applied aspects of the approach need to be further investigated before it can be widely implemented in clinical practice.

## Introduction

Large mechanical destabilizing disturbances during walking (such as slips and trips) lead to most falls among community-dwelling older adults (1–8). Interventions to reduce falls among older adults and clinical populations with balance impairment have received much attention in the literature, with multiple Cochrane reviews on the topic (9–14). Physical exercise is the most evidence-based approach for preventing falls, with challenging balance exercise among the most successful approaches (13, 15, 16). This aligns with the notion of task-specificity in exercise-based fall prevention (17–24), and the development of perturbation-based balance training (PBT).

Interest in the use of large mechanical perturbations as a method of preventing falls has steadily increased since the mid-2000s. In this period: Pai and Bhatt (18) presented a framework for using repeated slip perturbations to reduce slip-related falls; Grabiner et al. (19) presented evidence and theory on how the task-specific training of limiting trunk motion during slips and trips might reduce fall risk; Oddsson et al. (17) presented a balance training programme with a focus on training specificity, incorporating perturbations; and Mansfield et al. (25) published the first protocol for an RCT of PBT in older adults. Two subsequent large trials showed promising effects of PBT interventions on daily life fall incidence in older adults (26, 27) and another highlighted the clinical feasibility of this approach (28). Subsequent reviews and meta-analyses have further supported these encouraging results (29–33). More recently, a large, pragmatic RCT conducted in a clinical setting (34) and a smaller experimental trial (35) also reported positive fall-related outcomes. In contrast, a recent trial conducted in individuals with chronic stroke reported inconclusive results (36). Further RCTs of PBT are currently underway (37–41).

Despite the accumulating research on PBT, there is much still to be learned. Even so, practitioners are open to implementing PBT (42, 43) and desire more knowledge on the topic (43). In this review, we discuss some of the key concepts and common issues and questions around PBT. In doing so, we aim to provide a comprehensive synthesis of the current evidence on the mechanisms, feasibility, and efficacy of perturbation-based balance training for researchers and practitioners. We address this in two sections: “Principles and Mechanisms” and “Implementation in Practice.”

## Principles and mechanisms

### What is PBT?

**Figure 1:**
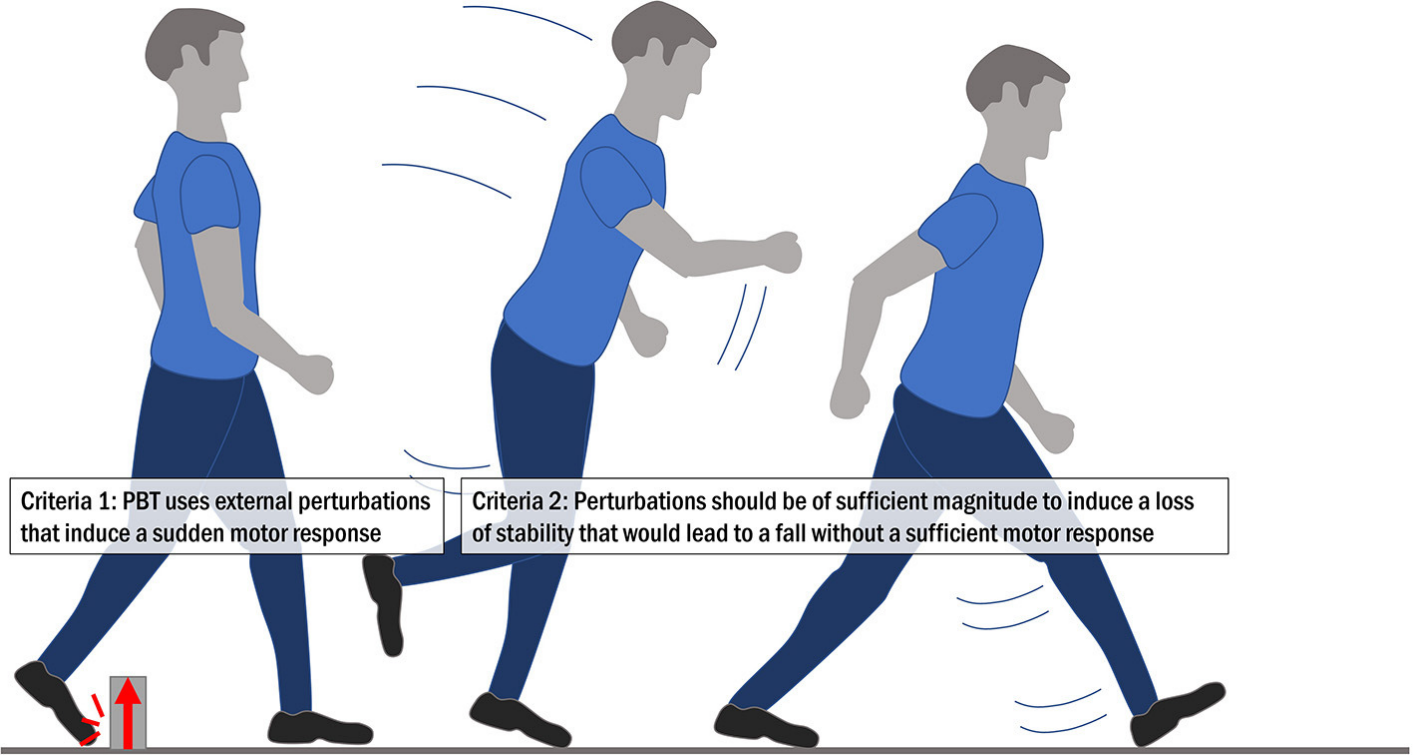
The two key criteria for perturbation-based balance training.

Various names for the same, or similar, training concepts to PBT can be found in the literature. These include reactive balance training, perturbation training, reactive step training and fall-resisting skills training, and there is not yet clear consensus on the best terminology. Here, we define PBT as *balance training that uses repeated, externally applied mechanical perturbations to trigger rapid reactions to regain postural stability in a safe and controlled environment*. The goal of PBT is to specifically target and improve the ability to recover stability in destabilizing situations like those that lead to falls in daily life. To meet this definition of PBT, the training should meet two key criteria (Figure 1): (1) the training should use external perturbations that induce a sudden motor response and, (2) these perturbations should be of sufficient magnitude to induce a loss of stability that would lead to a fall without a sufficient motor response (or use of the safety harness). Biomechanically, a loss of stability occurs when the position and motion characteristics of the center of mass exceed certain spatial and temporal limits relative to the base of support, whereby a fall becomes imminent without further action (44, 45). For this article and from a functional standpoint, we use the term balance as an umbrella term for all mechanisms and skills contributing to the maintenance of stability, with the term stability referring to the outcome or state (e.g., mechanically stable/unstable, fall/no fall).

### What is task-specificity in the context of PBT?

Our criteria for defining training as PBT, described in What is PBT?, specify that the training should use external perturbations that induce a sudden response and that are of sufficient magnitude to induce a loss of stability. In other words, if the perturbations used do not, (a) require a sudden response to compensate for the disturbance or, (b) lead to a loss of stability, we contend they are not sufficiently similar to the common causes of falls in daily life and are therefore, not task specific. For example, “internal perturbations” or instability induced by narrowing one's base of support or standing on an unstable wobbly surface are not considered PBT. A second consideration is that the method of perturbation delivery should be similar to common perturbations experienced in daily life. In this regard, pop-up obstacles on a walkway [like those used by Pavol et al. (46), Pavol et al. (47), Pijnappels et al. (48), Pijnappels et al. (49), Pijnappels et al. (50), Okubo et al. (51), Okubo et al. (52)] more closely simulate real life trips than a treadmill belt acceleration or deceleration [like those used by Sessoms et al. (53), Owings et al. (54), Grabiner et al. (55), McCrum et al. (56), for example], with cable-trip systems [e.g., as in Senden et al. (57), McCrum et al. (58) or Epro et al. (59)] lying in-between. While some studies suggest that the kinematics of the recovery actions triggered by treadmill-delivered perturbations are similar to more ecologically valid perturbations (53, 54), another study that directly compared treadmill belt accelerations with obstacle-induced trips while walking reported significant differences in trunk and stepping kinematics and their adaptations (60). As discussed in sections How does PBT lead to the retention and generalization of fall-resisting skills? and What technology is required for PBT?, the degree of similarity between the training and real-life trips and slips may have implications for the generalizability of PBT training approaches.

**Figure 2:**
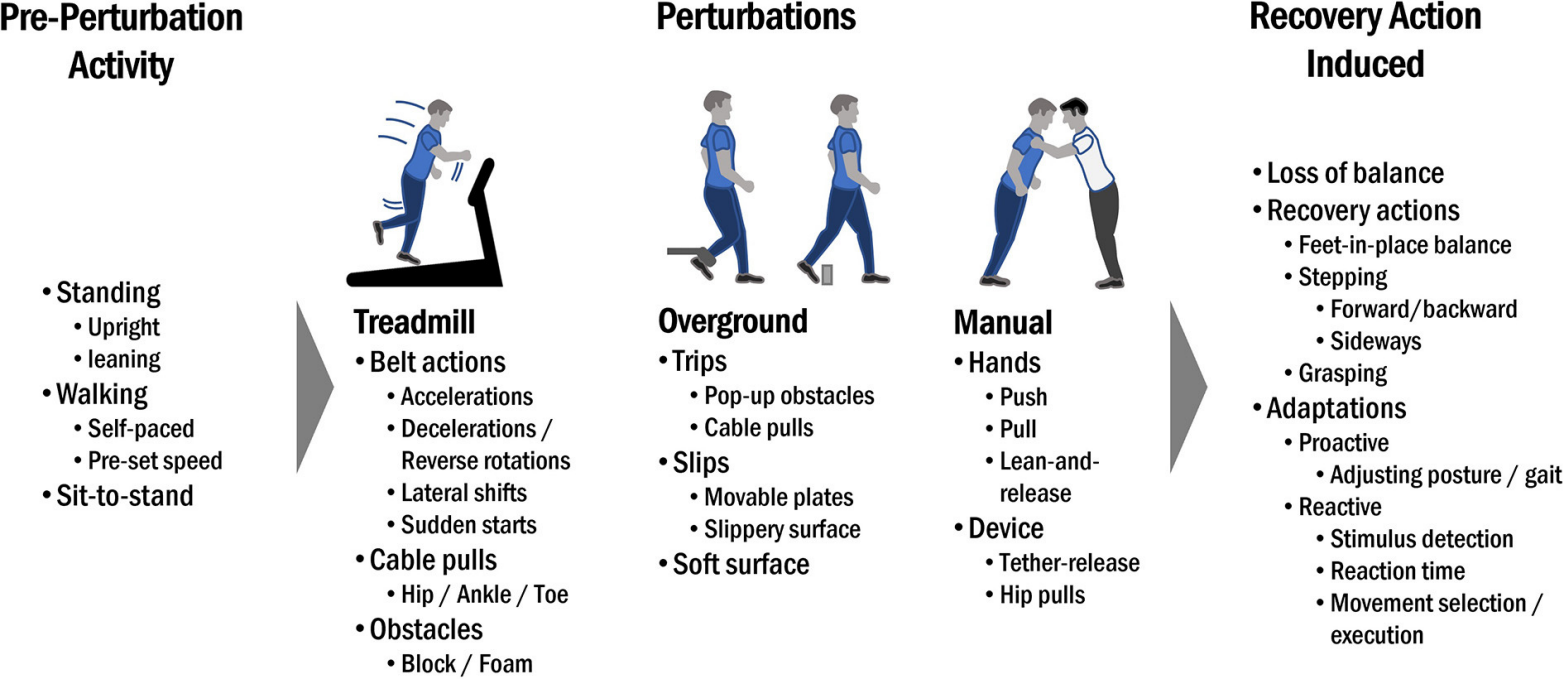
Examples of various task-specific elements to consider in PBT.

A third aspect of task specificity relates to whether perturbations are applied during standing, walking or other common movements (i.e., sit-to-stand transitions). As most falls in community-dwelling older adults occur during walking (1–6, 8, 61), this may be the most relevant task for PBT training for this group. However, frail older people, such as those living in residential care facilities, often experience falls during transitions (62–65), thus may benefit from standing and sit-to-stand perturbation training. Finally, due to the task-specific nature of PBT, training benefits may be restricted to improvements in dynamic and perturbed balance tasks with little or no transfer to less dynamic / static balance tasks (66–68). Some examples of various task-specific elements to consider are shown in Figure 2.

### How does PBT differ from other task-specific approaches to fall prevention?

Task-specific walking or balance training, even in the narrowed context of fall prevention, may take many forms. In addition to PBT these include: volitional step training with responses to various stepping targets, cues and constraints [for a review see: Okubo et al. (32)]; gait adaptability training using virtual or real obstacles [for example tasks see Geerse et al. (69) or Timmermans et al. (70)]; adapted forms of agility training [e.g., Donath et al. (71) and Lichtenstein et al. (72)]; and training with ongoing disturbances, simulating situations like uneven ground (73–75). Our criteria for PBT described above, however, distinguish PBT from these complementary approaches, in that regardless of how the perturbation is delivered (trip, slip, push, pull, to the trunk, to the foot, etc.), the participant must quickly identify and respond to a ‘sudden' perturbation. In contrast, in the other approaches, changes in the environment can be perceived prior to contact or the response is to a cue separate from a loss of stability. During PBT, the “cue” *is* destabilization, detected by the sensory systems, which triggers rapid stability-recovery responses. PBT facilitates sensorimotor adaptations in these stability-recovery responses through trial-and-error practice. Coupled with the criteria that PBT triggers a sudden response is the requirement that the perturbation causes a loss of stability. This element is conceptually similar to the definition of “challenging balance training” in previous reviews [e.g., balance training including two or three of the following criteria: movement of the center of mass; narrowing of the base of support; minimizing upper limb support (76)]. However, during PBT, usually conducted with a safety harness, participants' stability can and should be further challenged so that destabilization *always* occurs. This is also distinct from volitional step training and gait adaptability training, where appropriate stepping behavior to *avoid* stability loss is trained. Practically, in PBT, this sudden response following a loss of stability during standing or walking usually manifests as reactive stepping or reaching (when an appropriate support is available), as described by Maki and McIlroy (77) as a change-in-support strategy. If participants cannot retain their stability following a perturbation, they are caught by a safety harness or therapist. Such events form part of the sensorimotor skill learning, although whether complete recovery failure is necessary for successful intervention is currently unclear. There are also practical considerations that may affect how perturbation intensities leading to these failures are administered. These issues are addressed in sections What is the dose-response relationship for PBT?, How can anxiety be alleviated during PBT? and What is the evidence for PBT in clinical populations. Finally, while muscle strength training can't be considered a task-specific fall prevention intervention, strength training targeting functionally relevant muscle groups and actions could potentially be used in conjunction with PBT. One RCT (35) reported that PBT combined with hip muscle strengthening may further improve stepping performance and reduce daily life falls compared to PBT alone or strength training alone. This may suggest possible synergistic benefits of using PBT and targeted strengthening approaches. However, another study found no synergistic effects of PBT and training of plantar flexor muscles stability following trip perturbations (59), thus further investigation into such combined approaches is required.

### What are the mechanisms for PBT improving fall-resisting skills?

Early research demonstrated that a single session of repeated-perturbations (such as slip- or trip-like perturbations) results in acquisition of fall-resisting skills through implicit learning (without instruction) (78–81) across age-groups (young and old) (78) and tasks (standing, sit-to-stand transitions and walking) (82). In such single training sessions, the reduction of “in-task” falls can occur rapidly, i.e., in three–five trials (83). These improvements in recovery are associated with rapid improvements in both the feedforward/proactive control of stability (anterior shift of the center of mas) (83, 84) and the provision of proper limb support against collapse (78, 85–87), reflected in the form of improved recovery stepping responses, both during stance and walking perturbations. Depending on the perturbation type, the control of stability and limb support is achieved *via* changes in kinematic parameters such as recovery step length, trunk angle and velocity resulting from changes in neuromuscular output (88–90).

Motor adaptations, like those induced by PBT, may be predictive or reactive in nature (91–94). Predictive adaptation to a perturbation utilizes prior experience and knowledge of the upcoming perturbation in a feedforward manner to proactively adjust locomotor control and output (e.g., modifications of the base of support and/or center of mass position). Predictive adaptation can reduce the impact of a perturbation, reducing the magnitude of the required balance recovery response (95). Reactive adaptation, conversely, is a change in the motor responses to an unexpected perturbation. Reactive adaptation can manifest as: earlier detection of the perturbation or stability loss and faster stability recovery initiation (96, 97); optimization of motor programmes for stability recovery including facilitation and suppression of functionally relevant and irrelevant reflexes and reactions, respectively (98–100); and altered coordination in skeletal (especially weight bearing) muscles for rapid motor actions (18, 88, 94, 101–103). As discussed in section How does PBT differ from other task-specific approaches to fall prevention?, only PBT aims to improve the reactive stability recovery responses to destabilizing perturbations, as opposed to other task-specific approaches (e.g., gait adaptability training) that target predictive adaptations only. However, predictive adaptation, to some extent, is likely inherent in most PBT programmes (92). As such, PBT programmes should consider, and possibly monitor and account for, the role and influence of predictive adaptation, since it might reduce the impact of the administered perturbations and reduce generalization effects (31, 104, 105). Indeed, it has been demonstrated that perturbation impact might be significantly reduced if participants are aware they might encounter an unspecified hazard that may perturb their balance (105–107). Okubo et al. (108) also found that predictive adaptations are less readily observed when perturbation type, location and timing are unpredictable. However, other studies have shown that awareness of upcoming perturbations (109) or even observation training (watching videos of the perturbation task) (110) can lead to predictive adaptations but their effects were not comparable with those from actual physical experience of the perturbations.

### How does PBT lead to the retention and generalization of fall-resisting skills?

Promising results from early studies using overground slip perturbation training revealed that the skills acquired during a *single* repeated-slip training session can be retained for up to a year by developing protocols incorporating random practice [contextual interference (111–113)] and overlearning [continued task practice after reaching a success criterion (114–116)] *via* high intensity training (24 repeated slips) among healthy (young and older) adults (86, 117). Several other studies have subsequently shown good retention after exposure to repeated perturbations in healthy young adults (84, 86, 118, 119), older adults (59, 119), people with stroke (120–123) and Parkinson's disease (124–126). In terms of training dose, studies have included single sessions (117–119, 124, 127) and multiple sessions (59, 122, 123, 128) and retention intervals from as short as 30 min to up to 1.5 years post intervention.

A vital function of the central nervous system is its ability to apply motor adaptations obtained in one situation to a different situation. The central nervous system can generalize response adaptations to similar perturbations to an untrained limb (56, 129–131); untrained tasks [e.g., gait-slip to sit-to-stand slip (82)]; untrained contexts [e.g., moveable platform to vinyl floor (132–135)]; and to different perturbation types [slips to trips (136) and waist pull perturbations to treadmill slips (137), though minor interference has also been reported (81, 136)]. Generalization between contexts (treadmill to overground slips) may also be retained over longer periods (138). Based on evidence from locomotor training studies it is postulated that when an acquired internal representation is more general (i.e., not specific to certain effectors, environments or tasks) more motor transfer will ensue (139–143). This postulation seems applicable for PBT as well for fall prevention.

In summary, most reports indicate a positive transfer of adaptations between different conditions of the same perturbation, i.e., from treadmill gait-slips to a ‘novel' overground slip, or from training gait-slips on a moveable platform to an untrained slip on an oily surface (97, 133, 135, 144–146). However, several recent investigations have shown that improved balance skills resulting from repeated exposure to trip-like perturbations does not transfer to the recovery response to a similar large mechanical perturbation in the anterior direction (60, 119, 147). Critical components in neuromotor control (e.g., module composition and time-coordinated recruitment of motor modules) due to different neuromechanical task constraints (e.g., muscle activity patterns and body dynamics) may discriminate between perturbation types, possibly explaining the discrepancy between findings for generalization of adaptations from repeated gait perturbation exposure. Thus, although generalization is possible within the human stability control system, it may require a certain degree of similarity, if not consistency, between tasks which may be determined by factors other than shared limb mechanics. A recent study investigated potential factors limiting inter-task generalization within the stability control system (147). Differences were detected in the synergistic spatiotemporal organization of muscle activations indicating a diverging modular response to different perturbations, seemingly covered by the same main balance skill (i.e., rapid stepping). Hence, it may be argued that the transfer of adaptations in stability control between different balance tasks may be influenced by differences in muscle synergies in the perturbation recovery responses. Thus, while generalization of adaptation is in principle possible within the human stability control system, it seems limited if neuromotor factors discriminate perturbation responses in different motor tasks e.g., discrepancies in the spatiotemporal organization of the motor system between balance tasks (147).

### What is the dose-response relationship for PBT?

For a training protocol to be clinically accepted and implemented, the training dose-response relationship in addition to the training effect needs to be established (21, 148). A training dose can be varied by altering the intensity of the perturbation (making it more challenging), the amount of practice per session (increasing the number of perturbations) or the number of training sessions provided (148, 149).

For overground slip perturbations, earlier studies showed that a high practice dose (in terms of intensity) provided in a single session led to significant retention over the longer-term (4-6 months) (117, 128). Increasing the session frequency in terms of providing a booster dose did not lead to greater retention in younger adults (86, 150) but did so in older adults (128). However, increasing intensity, frequency and duration of such protocols could also have disadvantages such as activity-induced fatigue and reduced participation, particularly in certain clinical populations with significant health issues and balance impairments (151, 152). Another alternative for those unable to tolerate a high dose within a single session is to provide more sessions with fewer training trials or min per session (152). For example, studies have shown that a single slip exposure administered in separate, frequent sessions can induce lasting effects within the same environment (i.e., laboratory) (80, 150). No studies have examined dose effects for overground trip perturbations (148).

Several studies involving young adults, older adults and people with stroke have used different practice durations per session and number of sessions in their studies using treadmill belt perturbations. The number of trials per session have ranged from 11 to 80 and number of sessions have ranged from 1 to 24 (144, 145, 149, 153, 154). Retention periods have ranged from 30 min (144, 153, 155) to 6 months (138, 154).

There is a clear need for further dose-response studies (in particular, for the more clinically applicable treadmill-based protocols) to examine retention and generalization of the adaptations made, as the optimal dose for within-session or within-training programme adaptation may not necessarily be the same as the optimal dose for long term retention and generalization. Further, most studies have used only a single type of perturbation direction which may result in the limited real-life generalization observed. More studies are needed to examine the effect of bidirectional or multidirectional perturbation training on longer-term retention and generalization. Lastly, the type of perturbation training that yields maximum efficacy also remains unknown. These gaps are important to fill to provide recommendations to clinicians and develop clinical practice guidelines.

## Implementation in practice

### What are the primary safety issues in PBT?

PBT requires additional safety measures compared to conventional balance training. In this regard, safety harnesses are often used when administering large external perturbations. The benefit of a safety harness is that the participant can move in an almost unrestricted manner, and the therapist can focus on training delivery, with the assurance that any unsuccessful balance recovery will be safely arrested by the harness. Many different options are available, ranging from a fixed harness which can be attached to the ceiling in the middle of the exercise room or above a treadmill, or ceiling rail system harnesses which enable the wearer to move freely through a room. A portable/movable support frame is another option if appropriately certified for supporting a participant's body weight and does not interfere with reactive stepping responses. Harnesses also need to be well-fitted and comfortable to prevent harness-induced bruising and soreness after training.

Few adverse events from PBT training have been reported in the literature. Most studies report no or relatively minor adverse events such as soreness at the contact points between the body and the harness or muscle soreness (156–160). In 12 RCTs (20, 36, 52, 97, 122, 159, 161–165) summarized by Mansfield et al. (166), pain and delayed onset muscle soreness were the most commonly reported adverse events (16.4% of participants), with no severe adverse events reported in these trials. One other study reported 6 mild to moderate adverse events related to lateral waist-pull perturbations, including knee pain and groin injury, although the authors stated that this perturbation approach was generally well tolerated by the participants (35). Muscle soreness during or after training cannot be entirely prevented but may be decreased by adjusting training intensity for each individual. If an individual experiences a fall into the safety harness, follow-up assistance is often required to help them regain their stability and composure.

When working with less intensive external perturbations, such as therapist-applied perturbations, training is possible without additional safety equipment. However, it is crucial that both the therapist and patient know their limits and having a second therapist present to provide stability support is advised. Transfer belts can also assist the therapist apply perturbations as well as support their patients as required.

### How can anxiety be alleviated during PBT?

Anxiety and fear about upcoming perturbations and/or falling is a practical challenge in PBT (167). In their overview of 12 RCTs of PBT, Mansfield et al. (166) noted that about 5% of the included participants reported PBT-related fear or anxiety (some of which withdrew for this reason) and a more recent meta-analysis confirms that anxiety and fear occur more frequently in PBT than in control interventions (33). Anxiety during training is higher in older adults compared to younger adults and increases with greater uncertainty about the upcoming perturbations (51). In one study, older adults reported higher anxiety during PBT on a treadmill compared to PBT on an overground walkway (60). The authors suggest that this higher anxiety may have been due to unfamiliarity with treadmill walking and the elevated surface of the treadmill. Anxiety is higher in those with poor reactive balance, but heightened anxiety can also impair reactive balance control *via* delayed, more rigid and/or (poorly adapted) startle responses (168–170), and thus should be minimized for a better training outcome. Monitoring of anxiety levels using a custom 5-point scale and adjustments of training intensity (e.g., 5–10% reduction in gait speed) have been effective in easing anxiety during reactive balance training using overground trips and slips (52). Interviews with participants who underwent PBT using an instrumented treadmill system (171) revealed that while some participants experienced anxiety during training, most described feeling a “good kind of nervousness” during training, rather than anxiety. Participants that reported being initially anxious often found that their anxiety diminished or resolved after the first training session when they had experienced PBT and were confident they could recover from the perturbations, a finding also reported by Jagroop et al. (167). The presence of safety equipment (especially a safety harness), and ensuring participants are heard and informed during the training sessions have been identified as important factors that mitigate anxiety (171). In cases where sufficiently large destabilizing perturbations increase anxiety and possibly prompt withdrawal, it may be prudent to administer training intensities that are less threatening until anxiety is reduced. This may reduce the effectiveness of the initial training period and may not qualify as PBT as per our definition but may retain patients in training and allow them to become more comfortable with the training regime and take part in higher intensity PBT in subsequent trials. Uncertainty about the timing, location, type or direction of perturbations (in situations in which these are modifiable options) can also be gradually increased congruent with the comfort and performance levels of participants.

### What is the evidence for PBT in clinical populations?

To date, PBT has been studied primarily in healthy community-dwelling older adults. However, there is also emerging evidence for the effectiveness of PBT in ‘high risk' older adults (for example assisted living residents, or older adults with a history of falls or balance problems), and people with Parkinson's disease, stroke and multiple sclerosis (121–123, 156, 158, 159, 172–176). PBT trials have also been conducted in people with chronic obstructive pulmonary disorder (152) and incomplete spinal cord injury (165), but due to limited findings will not be discussed in detail in this article. Previous reviews (29, 30) showed significant fall reductions in community-dwelling older adults, frail/high-risk older adults and people with Parkinson's disease and stroke following PBT. PBT has also been found to improve perturbation recovery measures (156, 159, 160, 177) and some studies have reported improvements in clinical balance tests such as the Berg Balance Scale in people with Parkinson's disease (173, 174, 178). However, while there appears to be interest in the potential for PBT to improve a broad range of gait and balance measures in clinical populations [see reviews of Hulzinga et al. (179), Coelho et al. (180)], as outlined in section What is task-specificity in the context of PBT? and How does PBT differ from other task-specific approaches to fall prevention?, the effects do not necessarily generalize to less-reactive balance and gait measures. To our knowledge, no current studies in clinical populations have reported non-responders in terms of adaptation of the stability recovery response to PBT. However, on an individual level, those who cannot tolerate being exposed to perturbations (due to, for example, anxiety or pain) may not be able to benefit from PBT immediately, and perhaps initially require more basic balance training.

There are some important factors to consider before applying PBT in less able populations. First, decreasing training intensity to an acceptable level for the participant may mean that the total training volume is increased to compensate. Second, frailer people may require a walking aid in daily life. To our knowledge, no studies have focused on the feasibility of using walking aids during PBT, but we hypothesize that the use of a full-body harness with partial bodyweight support may enable PBT for these people. Future studies may focus on this gap in knowledge.

### What technology is required for PBT?

Several mechanical perturbation systems can evoke the balance disturbances required for PBT and trigger error-driven motor learning in the control of postural balance. As there is a growing body of evidence suggesting both the efficacy and efficiency of PBT for improving fall resisting skills, there is also a need to further develop devices which are capable of mimicking disturbances experienced during daily-life mobility in clinical settings.

An ideal system for training reactive balance recovery should be capable of applying unpredictable mechanical perturbations of different magnitudes and directions and/or types at pre-specified timepoints that elicit a loss of balance and thus mimic near-fall situations in a safe, controllable environment (31, 181, 182). This system should also be able to measure the participant's stability and stability recovery to facilitate assessment and personalized training.

Several perturbation systems have been used to disturb stability during walking, including floor obstacles in both overground (46, 51, 113, 183–186) and treadmill setups (181, 187), unexpected surface compliance changes [overground; (188)], overground slips or surface translations (133, 189, 190), cable or rope trips both in overground (191, 192) and treadmill setups (57, 88, 193–196), as well treadmill-based belt speed changes (53, 55, 61, 118, 197–199), platform translations or tilts (200) and waist/torso pushes and pulls (137, 201–204). Several commercially available systems are also available (e.g., BalanceTutor, ActiveStep, C-Mill React). It is important to highlight that no system is without its limitations. For example, overground setups suffer from the limitation of limited walkway length and that the location of the perturbations may not be entirely unpredictable (31), though this limitation can be, at least partly, addressed by including multiple possible perturbation locations [see, for example, (108)]. The obvious advantage of the treadmill in comparison to such overground setups is that predicting when a perturbation will be applied is more difficult, as there is no location-based reference point (31), which ensures that predictive adjustments in in anticipation of perturbations are reduced [though not necessarily completely absent (123, 155)]. However, walking on a treadmill can provide additional challenges in some populations at increased fall risk, due to lack of familiarity and the requirement to maintain a specific speed [walking speed can be instantaneously adjusted in an overground setup but maintaining it provides an additional challenge during perturbed treadmill walking (205)]. Another inherent limitation in some setups is that the perturbations themselves may not strictly mimic common causes of falls like slips and trips (60) despite the subsequent recovery mechanics being suggested to be similar (53, 54) (see also Figure 2 in section What is task-specificity in the context of PBT?). A recent study reported that adaptations observed with repeated treadmill belt accelerations did not transfer to obstacle-induced trips while walking (60). However, it is not currently known if and how this affects transfer to daily life situations. Another factor that should be considered is the ease with which PBT dose can be altered. The number of perturbations and training sessions can be easily manipulated but not all systems can provide a wide range of perturbation magnitudes which is critical to ensure that participants are safely and sufficiently destabilized, even late in their training. This is of particular relevance for the conceptualization of fall prevention interventions in clinical settings because the hypothesis of a non-linear dose-response relationship (148) implies that adaptation may not be directly related to the applied practice dose and that a dose threshold exists beyond which any additional stimuli may not induce further changes.

In summary, based on current evidence, we believe that the primary factors for a successful PBT system are that it can; (a) administer perturbations that are difficult for participants to predict (in time of onset but perhaps also in body location, mode or magnitude of application); (b) suddenly destabilize participants with these perturbations; and (c) easily adjust the magnitude of perturbations.

Despite the potential advantage of using such systems to destabilize participants and create near fall situations, the costs associated with the equipment, as well as the expertise required to operate PBT systems may hinder their application in clinical settings. Thus, there is a need to develop alternative, feasible PBT programmes that do not require these devices. Therapist-applied perturbations, as described above, are the natural alternative and can be easily applied if appropriate safety measures are followed. However, managing the training and perturbation dosage may be problematic due to the perturbations being more predictable and the intensity of therapist-applied perturbations being less precise. Such limitations, however, do not discount the potential effectiveness of this approach when they constitute the only feasible option in at least the short term. For a useful resource on the therapist-applied perturbation approach, we refer readers to Mansfield et al. (166).

### Is PBT appropriate in at-home, group or semi-supervised settings?

The application of PBT in home or group settings has been little investigated to date. Clearly, it is not safe to apply large external perturbations, with the possibility of an unsuccessful balance recovery in the absence of a safety harness. Smaller perturbations however, such as therapist-applied perturbations, may be applied in home and group settings. For example, Oddsson et al. (17) successfully applied perturbations in a group setting through training in couples with partner or therapist-applied perturbations.

As discussed above, it is crucial the participant feels safe during training, and everyone involved know their limits. Portable safety equipment, such as a transfer belts, can assist the therapist apply the perturbations as well as support patients during training. However, if an appropriate training stimulus cannot be reached this way, transferring the training to a one-on-one basis, or using more specific equipment should be considered. Future studies are necessary to elucidate the feasibility of PBT in a group or semi-supervised setting.

## Recommendations

Taking the previous sections into account, several recommendations for both research and clinical application of perturbation-based balance training can be made.

### Research

Studies are required to:

- Determine optimal training doses and the potential effects of repeated training or booster sessions.
- Identify the relative contribution of different aspects of training dose (e.g., perturbation impact, perturbation training intensity (displacement, velocity, acceleration settings), perturbation number, training session number) to the training effects.
- Compare the effects of different laboratory-based PBT methods with respect to stability outcomes and daily life fall prevention.
- Further elucidate and compare the criteria by which adaptations gained by training one type of perturbation transfer to other similar perturbations (e.g., between legs, a movable plate to a slippery floor; see How does PBT lead to the retention and generalization of fall-resisting skills? above).

### Clinical application

There is a need to:

- Develop effective, affordable and clinically feasible methods for applying perturbations.
- Conduct feasibility studies to explore opportunities and barriers for implementation.
- Determine strategies to alleviate anxiety in participants undertaking PBT to ensure clinical feasibility.
- Identify which clinical populations with balance impairment benefit from PBT
- Elucidate PBT dose-response relationships in these populations.

Finally, it is worth highlighting that there have been only a few randomized controlled trials with sample sizes large enough to have statistical power to evaluate the role of PBT in reducing daily life falls. Lurie et al. (34) with their multicenter pragmatic (non-standardized protocol based on therapist judgement) trial is the largest. This 12-month trial included 187 participants (of 253 allocated) who received PBT and 190 (of 253 allocated) participants who received standard balance training. Once some of the issues relating to training and practice mentioned above have been further elucidated, we recommend large, definitive trials following CONSORT guidelines are conducted. In the meantime, we recommend that studies on PBT collect and report prospective falls data as secondary outcomes to assist future meta-analyses. Template forms for collecting falls information following recommendations by Lamb et al. (206) and Lord et al. (207) can be downloaded at http://doi.org/10.17605/OSF.IO/HMJEF (208).

## Conclusions

Perturbation-based balance training is a promising approach to fall prevention. This task-specific training of balance using repeated exposure to sudden perturbations may present a paradigm shifting approach that may improve effectiveness and efficiency of a fall prevention exercise intervention. However, several fundamental and applied aspects of the approach need to be further investigated before this approach can be widely implemented in clinical practice.

## Author contributions

CM and YO: conceptualization, project administration, writing-original draft, and writing-review and editing. TB, MG, and KK: writing-original draft and writing-review and editing. MR: writing-review and editing. SL: conceptualization and writing-review and editing. All authors contributed to the article and approved the submitted version.

## Conflict of interest

The authors declare that the research was conducted in the absence of any commercial or financial relationships that could be construed as a potential conflict of interest.

## Publisher's note

All claims expressed in this article are solely those of the authors and do not necessarily represent those of their affiliated organizations, or those of the publisher, the editors and the reviewers. Any product that may be evaluated in this article, or claim that may be made by its manufacturer, is not guaranteed or endorsed by the publisher.
